# OFIDA: Object-focused image data augmentation with attention-driven graph convolutional networks

**DOI:** 10.1371/journal.pone.0302124

**Published:** 2024-05-02

**Authors:** Meng Zhang, Yina Guo, Haidong Wang, Hong Shangguan

**Affiliations:** School of Electronics and Information Engineering, Taiyuan University of Science and Technology, Taiyuan, Shanxi, China; University of California Los Angeles, UNITED STATES

## Abstract

Image data augmentation plays a crucial role in data augmentation (DA) by increasing the quantity and diversity of labeled training data. However, existing methods have limitations. Notably, techniques like image manipulation, erasing, and mixing can distort images, compromising data quality. Accurate representation of objects without confusion is a challenge in methods like auto augment and feature augmentation. Preserving fine details and spatial relationships also proves difficult in certain techniques, as seen in deep generative models. To address these limitations, we propose OFIDA, an object-focused image data augmentation algorithm. OFIDA implements one-to-many enhancements that not only preserve essential target regions but also elevate the authenticity of simulating real-world settings and data distributions. Specifically, OFIDA utilizes a graph-based structure and object detection to streamline augmentation. Specifically, by leveraging graph properties like connectivity and hierarchy, it captures object essence and context for improved comprehension in real-world scenarios. Then, we introduce DynamicFocusNet, a novel object detection algorithm built on the graph framework. DynamicFocusNet merges dynamic graph convolutions and attention mechanisms to flexibly adjust receptive fields. Finally, the detected target images are extracted to facilitate one-to-many data augmentation. Experimental results validate the superiority of our OFIDA method over state-of-the-art methods across six benchmark datasets.

## 1 Introduction

Data augmentation (DA) is an essential technique in machine learning and data analysis. By augmenting the labeled training data with greater quantity and diversity, data augmentation effectively tackles the issue of limited data availability, thereby preventing the model from memorizing specific instances and promoting better generalization to unseen data.

Numerous image data augmentation techniques have been studied to address the problem of limited or scarce data. Image manipulation [1, 2], image erasing [3–5], and image mix [6, 7], are some of the basic image data augmentation methods. Advanced image data augmentations include auto augment [8, 9], feature augmentation [10, 11], and deep generative models [12, 13].

These methods contribute to improving training data diversity and quality, but face challenges. Firstly, traditional methods struggle to capture the complexity of real-world objects accurately [14]. Secondly, image erasing techniques may unintentionally remove crucial details or distort image structures, resulting in information loss [15]. Additionally, image mix and auto augment methods can generate unrealistic pixel values or patterns, deviating from real image distributions and affecting visual quality [14]. Lastly, GAN-based approaches require large-scale datasets and significant computational resources, posing constraints on data and computational requirements, limiting their feasibility in some applications [16].

To address these limitations, the aim of this paper is to propose a novel image data augmentation method that can preserve important target regions and simulating real-world scenes and data distributions, while providing more diverse and precise image samples to improve the performance of machine learning models in learning data features and variations.

A potential approach to aforementioned problem is to accurately identify target regions in images and perform precise and diverse one-to-many data augmentation that separate detected objects from the original images. However, this approach may face several challenges, such as low detection accuracy of typical object detection algorithms, difficulty in detecting small objects, and limited sensitivity to object occlusion, density, and shape variation. In addition, existing image data augmentation methods often are not easy to generate entirely new samples or capture the full complexity of real-world objects, and may risk removing important details or introducing unrealistic results. The aforementioned limitations can be referred to as an object-focused image data augmentation (OFIDA) problem. This paper comprehensively addresses the problem and our contribution is two-fold:

1. OFIDA model: A new model for the OFIDA problem is proposed with the aim of performing precise and diverse augmentations that preserve important target regions and improve the simulation of real-world scenes and data distributions. Specifically, the model identifies object regions in images and applies a one-to-many data augmentation strategy that separates detected objects from the original images. This ensures that object regions are accurately preserved while also enabling the generation of diverse samples.
2. OFIDA algorithm: Based on the OFIDA model, we further introduce the OFIDA algorithm, which is based on the following core concept:
  (a) We introduce the OFIDA algorithm, which involves initial object detection to identify target regions. A unique one-to-many data augmentation strategy is then applied, separating detected targets from original images for accurate preservation and diverse samples.
  (b) To achieve more precise identification and classification of target regions, we introduce the DynamicFocusNet algorithm based on a graph structure. This approach addresses the limitations of current object detection methods that solely rely on basic convolutional layers for classification, effectively resolving issues related to inaccurate classification and recognition(elaborated further in Section 4).
  (c) We extensively analyze our approach on large-scale public datasets including CIFAR10, CIFAR100, ImageNet, PASCAL VOC, CITYSCAPES, and MS-COCO 2017. Experimental findings consistently demonstrate the superior performance of our proposed methodology compared to state-of-the-art benchmark methods. This validation underscores the efficacy of OFIDA across a diverse range of computer vision tasks.

The paper is organized as follows. Section 2 describes the related work. Section 3 formulates a new mathematical model for the OFIDA problem. Section 4 presents our proposed algorithm for the problem of OFIDA. Section 5 shows numerical results. Section 6 concludes the paper and draws potential future research directions.

## 2 Related work

### 2.1 Image data augmentation

Image data augmentation techniques have become a critical component in enhancing the generalization capability and performance of data-driven inference in recent years, particularly in fields such as computer vision (CV) [17, 18]. Image data augmentation enables the creation of realistic variations of existing data, thereby increasing the amount of training data without requiring additional ground-truth labeling efforts. Generally, image data augmentation techniques can be classified into two main branches: basic and advanced image data augmentations. The former encompasses fundamental techniques, while the latter encompasses more complex ones. Each image data augmentation method is described below.

#### 2.1.1 Basic image data augmentations

Basic image data augmentations can be further divided into three main categories: image manipulation, image erasing, and image mix.

Image manipulation is a commonly used technique in computer vision tasks. Basic image manipulations like rotation, flipping, cropping, and direct image transformations are valid only if they are compatible with the data distribution of the images being manipulated. However, some basic manipulations like translation and rotation can cause loss of image content or moving some parts out of the boundary, known as the padding effect. Image erasing is becoming popular, and techniques like Cutout [1], Hide-and-Seek [2], Random Erasing [3], GridMask [4], and FenceMask [5] are some examples of it. Image mix data augmentation is another popular technique, and techniques like Fmix [6], AugMix [7], and ManifoldMix [19] have been proposed in this regard. Although these techniques can improve the performance of convolutional neural networks, they also have certain limitations and drawbacks. One major limitation is the risk of overfitting or poor generalization if the augmentation is too aggressive or introduces unrealistic features.

#### 2.1.2 Advanced image data augmentations

The field of computer vision has experienced a surge in interest in image data augmentation techniques in recent years, leading to the development of a variety of innovative methods for augmenting image data. Some of these methods include auto augment, feature augmentation, and deep generative models.

Various automated methods have been proposed to search for effective augmentation operations, such as RandAugment [9], KeepAugment [20], and OHL-Auto-Aug [21], but they have limitations in terms of their search space and computational cost. Additionally, feature augmentation has gained attention as an alternative to input space augmentation [22], with methods such as Moment Exchange [11], but it may require domain-specific knowledge to identify meaningful features. Deep generative models, such as GANs [23], can generate synthetic data, but evaluating the quality of generated data remains a challenge [24]. Conditional adversarial networks [25], can learn the mapping from input to output images, but they may struggle to handle complex and diverse image domains. StarGAN [12] and StarGAN v2 [13] have improved scalability and diversity across multiple domains, but may still suffer from domain shift issues.

### 2.2 Graph convolutional networks

Graph Neural Networks (GNNs) [26], which inherit the power of neural networks and utilize the structural information of graph data concurrently, have achieved remarkable success in various graph-based tasks [27–32], including node classification, graph classification, and graph generation.

Recent advancements in graphical architectures have greatly accelerated progress in multi-label image recognition. Li *et al*. [33] utilize a Graphical Lasso framework to model image-dependent conditional label structures. Li *et al*. [34] create a tree-structured graph in the label space using a maximum spanning tree algorithm. Additionally, Graph Convolutional Networks (GCNs) have shown remarkable capacity in various vision tasks. For instance, Chen *et al*. [35] employ GCNs to propagate prior label representations, such as word embeddings, and generate a classifier by replacing the last linear layer in a typical deep convolutional neural network such as ResNet [36]. Moreover, Chen *et al*. [37] utilize label annotations to compute a probabilistic matrix as the relation edge between each label in a graph.

In this paper, we develop a new object-focused image data augmentation (OFIDA) that tackles the challenges in accurately identifying target regions and generating diverse and precise image samples. Our proposed method integrates multiple algorithms, including an optimized attention mechanism, a dynamic graph convolutional network (D-GCN), a novel object detection algorithm called DynamicFocusNet, and a modified cropping technique, to enable one-to-many data augmentation.

## 3 Mathematical model

The aim here is to utilize a concept of one-to-many through object-separation in order to create a new data augmentation algorithm, known as object-focused image data augmentation (OFIDA). In this section, a mathematical model is presented for the OFIDA problem. To facilitate the description, the process is divided into three parts: feature extraction, classification and regression, and separation. The mathematical models for each part are introduced below.

**Feature extraction**. To begin with, feature extraction is necessary to detect objects in the input image, *F* represents the process of feature extraction, which transforms the input image **I** into the feature pyramid **F**_*l*_:

$$\begin{array}{c} F\left(\text{I}\right)=\{{\text{F}}_{l}{\}}_{l=1}^{L}, \end{array}$$ (1)

$$\begin{array}{c} \text{I}\in R^{H_{l}\times W_{l}\times C_{l}}. \end{array}$$ (2)

where **I** represents the input image, **F**_*l*_ represents the *l-th* layer feature map in the pyramid. the *l* represents an index ranging from 1 to *L* (*L* = 5). *H*_*l*_, *W*_*l*_, and *C*_*l*_ denote the height, width, and number of channels of the *l- th* feature map.

**Classification and regression**. After obtaining the feature maps *F*_1_, *F*_2_, …, *F*_*l*_, the feature maps are then utilized for classification and regression.

In the classification stage, each position (*x*, *y*) on each feature map **F**_*l*_ is divided into *i* anchor boxes, which can generate a candidate boxes of different scales and aspect ratios. Each candidate box is classified using the classification function, yielding the probability of belonging to each class. Specifically, for each position (*x*, *y*) on each feature map **F**_*l*_ and each candidate box **b**_*i*_:

$$\begin{array}{c} c_{\left(0\right)}={\text{P}}_{cls}^{l,ori}\left(x,y,i\right), \end{array}$$ (3)

where *c*_(0)_ represents the probabilities of the classification by a basic classification function ${\text{P}}_{cls}^{l,ori}\left(x,y,i\right)$. However, in (3), the classification function ${\text{P}}_{cls}^{l,ori}\left(x,y,i\right)$ may suffer from accuracy issues in complex scenes, especially in data augmentation tasks based on detection and separation, where high classification accuracy is crucial.

To overcome this drawback, we utilize a dynamic graph neural network with proposed content-aware attention module (CAAM) to further refine the classification results and obtain a new classification function:

$$\begin{array}{c} c_{\left(1\right)}={\text{P}}_{cls}^{l,gcn}\left(x,y,i\right), \end{array}$$ (4)

where *c*_(1)_ represents the probabilities of the classification by a improved classification function ${\text{P}}_{cls}^{l,gcn}\left(x,y,i\right)$.

Inspired by the concept of the averaging strategy, which serves to mitigate potential biases or errors and harmonize the performance disparities between the base model and the enhanced model, the ultimate classification outcomes, denoted as *c*, are derived.

$$\begin{array}{c} c=\frac{1}{2}(c_{\left(0\right)}+c_{\left(1\right)}). \end{array}$$ (5)

Next, the regression function R is utilized to regress the offset of each candidate bounding box relative to its anchor point. Specifically, for each position (*x*, *y*) on the feature map **F**_*l*_ and each candidate bounding box **b**_*i*_:

$$\begin{array}{c} \begin{array}{cc} \text{H} & =\Delta \text{b}+{\text{b}}_{i}\\ & =\text{R}\left({\text{b}}_{i}\right)⊙{\text{b}}_{i}+{\text{b}}_{i}. \end{array} \end{array}$$ (6)

where **H** denotes the regression vector of candidate bounding box **b**_*i*_ at position (*x*, *y*) on the feature map **F**_*l*_. The symbol ⊙ denotes the element-wise multiplication operation and position offset **Δb** is obtained by R(**b**_*i*_) ⊙ **b**_*i*_.

Non-maximum suppression algorithm (NMS) is used to filter all candidate bounding boxes based on their confidence scores, remove highly overlapped bounding boxes, and obtain the final detection results, NMS is expressed as follows:

$$\begin{array}{c} S_{i}=\{\begin{array}{cc} S_{i}, & \text{IoU}(\text{m},{\text{b}}_{i})<N_{t}\\ S_{i}(1-\text{IoU}(\text{m},{\text{b}}_{i})). & \text{IoU}(\text{m},{\text{b}}_{i})⩾N_{t} \end{array} \end{array}$$ (7)

where *S*_*i*_ represents the score assigned to each bounding box, reflecting its likelihood of containing the object of interest. The value of *S*_*i*_ is influenced by the particular algorithm employed and the chosen strategy for scoring. **m** represents the ground truth bounding box, **b**_*i*_ represents each candidate bounding box, and *N*_*t*_ is the set threshold. It can be observed that the score of the bounding box linearly decreases when the IoU score exceeds *N*_*t*_. IoU generally refers to the Intersection over Union ratio function between the candidate bounding box **b**_*i*_ and the ground truth bounding box **m**:

$$\begin{array}{c} \begin{array}{cc} j & =\text{IoU}(\text{m},{\text{b}}_{i})\\ & =\frac{{\text{b}}_{i}\cap \text{m}}{{\text{b}}_{i}+\text{m}-\left({\text{b}}_{i}\cap \text{m}\right)}. \end{array} \end{array}$$ (8)

where *j* represents the calculated value of IoU, which is a measure of overlap between two bounding boxes. It ranges between 0 and 1, indicating the extent of spatial agreement between the bounding boxes.

**Separation**. After the NMS algorithm, the bounding box with the highest score is selected as the final detection result, denoted as the target bounding box **t**_*i*_. For each target bounding box **t**_*i*_, a set of information can be obtained based on the classification score *c* and the candidate bounding box score *S*_*i*_, that is, **t**_*i*_ = {*x*_*i*_, *y*_*i*_, *w*_*i*_, *h*_*i*_, *c*_*i*_}, where (*x*_*i*_, *y*_*i*_) represents the coordinate of the upper-left corner of the target bounding box **t**_*i*_, *w*_*i*_ represents the width, *h*_*i*_ represents the height, and *c*_*i*_ represents the color of the target bounding box **t**_*i*_ for different classes. The separation function CP is defined as:

$$\begin{array}{c} {\text{I}}_{i}=\text{CP}(\text{I},t_{i}). \end{array}$$ (9)

where **I** refers to the input image, and **I**_*i*_ refers to the *i-th* cropped small image from the original image.

Ultimately, building upon (1) to (8), we propose a novel object detection algorithm known as DynamicFocusNet. It uses a dynamic graph neural network with proposed content-aware attention module (CAAM) and draws inspiration from the averaging strategy to improve the accuracy of detection results. Furthermore, we introduce a one-to-many data augmentation technique, referred to as object-focused image data augmentation (OFIDA), which utilizes (1) to (9) to classify, localize, and separate the target images.

## 4 Object-focused image data augmentation

**Fig 1:**
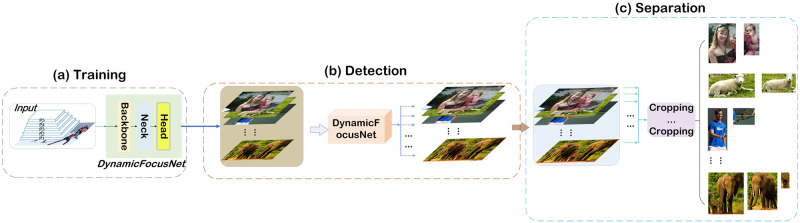
The working process of the OFIDA. Training DynamicFocusNet with the MS-COCO 2017 dataset to achieve accurate classification and localization of target images (a). Evaluating the performance of DynamicFocusNet using the MS-COCO 2017 test set (b). Utilizing the trained DynamicFocusNet to detect and localize target images in original images (c), and employing a cropping technique to accurately separate detected objects from original images (d), enabling precise one-to-many image data augmentation of samples.

In this section, we present an integrated multi-task algorithm in a two-step solution where object-focused image data augmentation (OFIDA) is performed to solve the problem in (9), as shown in Fig 1. The first step is to utilize the DynamicFocusNet algorithm to detect and locate the target regions within the images. The second step is to apply cropping technique to separate target regions, enabling precise one-to-many image data augmentation of samples.

**Ethics statement** The images presented in the figures are sourced from the publicly available MS-COCO Dataset [Dataset Link: https://cocodataset.org/], which is constituted by a diverse group of volunteers. The utilization of this dataset has been explicitly approved and authorized by the dataset providers for academic research purposes.

### 4.1 Proposed OFIDA algorithm

**Fig 2:**
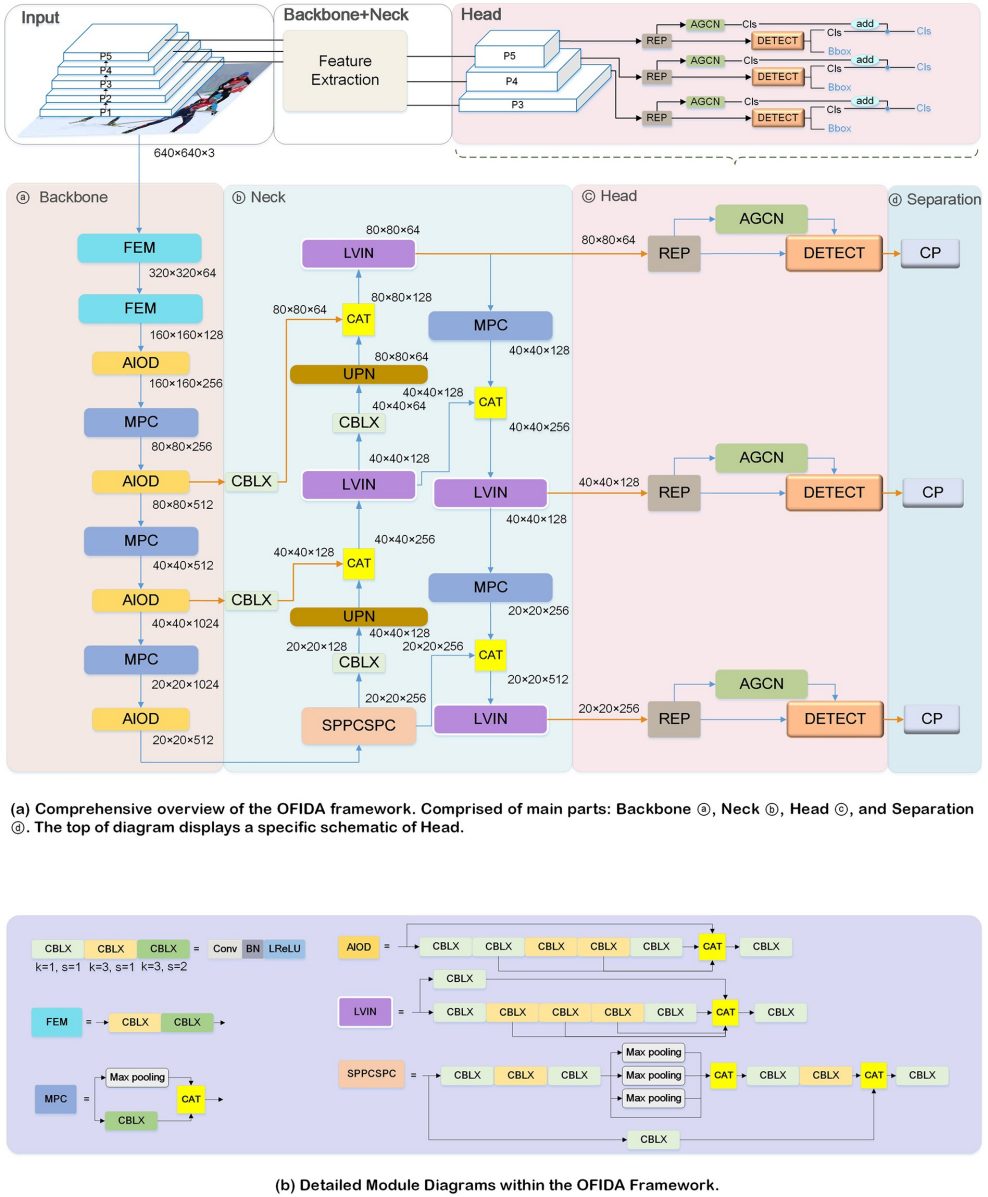
Integrated view of the OFIDA framework and its modules.

The OFIDA consists of four main components: Backbone, Neck, Head, and Separation, as depicted in Fig 2(a). Among them, Backbone, Neck, and Head constitute the DynamicFocusNet. In the following sections, we will provide detailed descriptions of each module, explaining their individual roles and functionalities within the framework.

**Backbone**. According to (1), our goal is to transforms the input image **I** into the feature pyramid **F**_*l*_. To achieve this, we develop a lightweight CSPNetX as our backbone network, which possesses advantages such as high efficiency, powerful feature extraction ability, and low GPU memory consumption. Fig 2(a) ⓐ a illustrates the architecture of CSPNetX, comprising three modules: the Feature Extraction Module (FEM), the Adaptive Internal-Depthwise-and-Output (AIDO) module, and the MPC module. The FEM and MPC modules are responsible for extracting features and performing downsampling operations, while the AIDO module serves as an efficient network structure that controls the shortest and longest gradient paths, enabling the network to learn more features and enhance its robustness. The backbone network performs feature extraction on the input image, generating multi-layer features at different scales, which are commonly referred to as a pyramid structure.

**Neck**. The neck network consists of three main components: Spatial Pyramid Pooling Cross Stage Partial Network (SPPCSPC), UPN, and Light-weight and Versatile Integrated Network (LVIN), as shown in Fig 2(a) ⓑ. The SPPCSPC module utilizes max pooling to acquire diverse receptive fields, enabling the DynamicFocusNet algorithm to adapt to images of various resolutions. The UPN module performs upsampling operations. The LVIN module represents an enhanced version of the AIDO module, incorporating techniques such as expand, shuffle, and merge cardinality to continually improve the module’s learning capacity while preserving the original gradient path. Through the integration of features from different levels and scales, the neck network seamlessly connects these features to the head.

**Fig 3:**
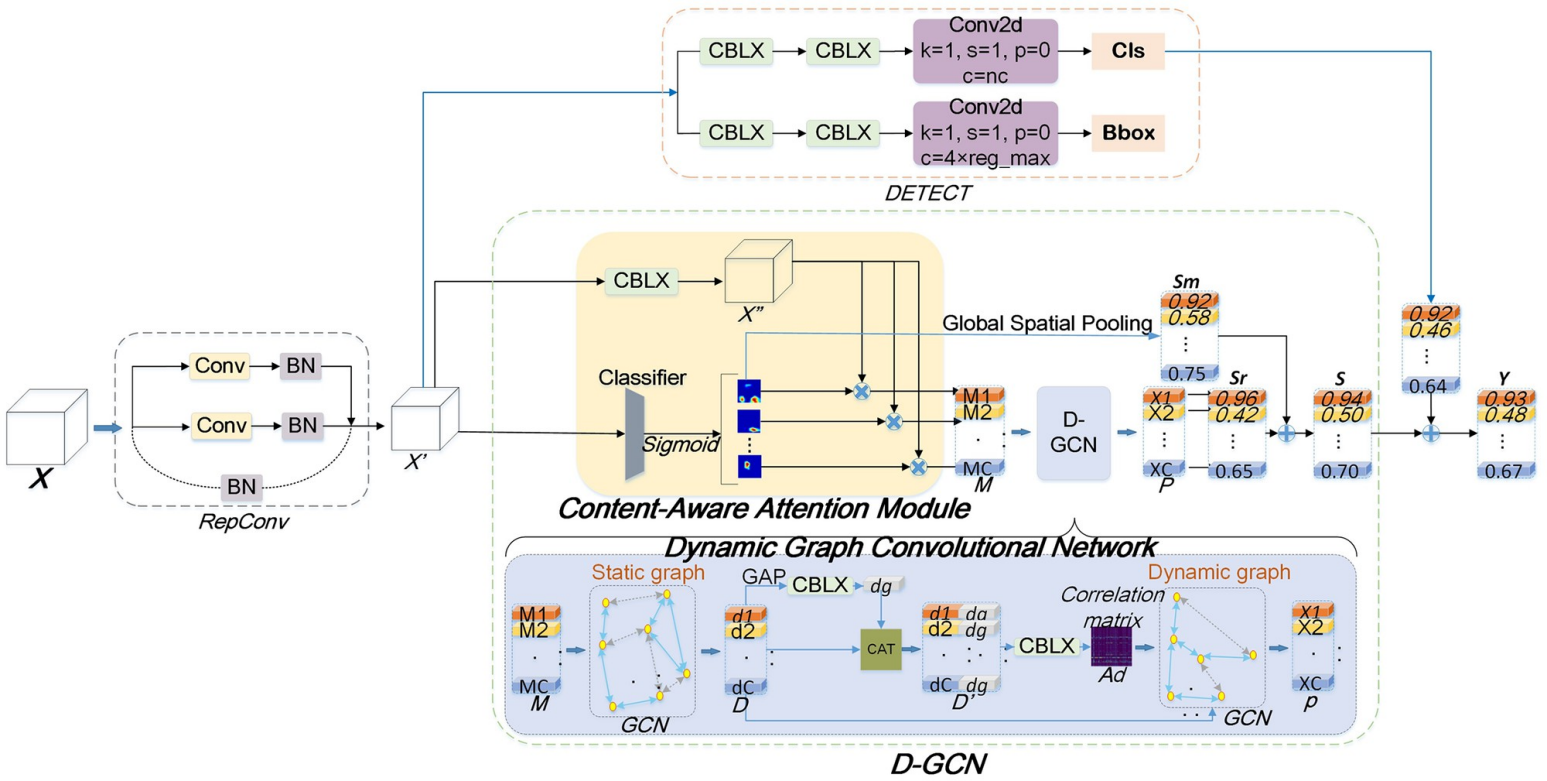
The framework of our head network. Given a feature map **X**, RepConv conducts parameter reorganization, resulting in **X**′. Then, the content-aware attention module (CAAM) separates content-aware category representations **M** from **X**′. The Dynamic Graph Convolutional Network (D-GCN) models global and local relations in **M**, generating a robust representation **P** with rich relational information across categories. Object detection is performed by DETECT on **X**′, producing classification scores **Cls** and bounding box regression results **Bbox**. Finally, the classification scores **Cls** are averaged with **S**, yielding the final scores **Y** for each category.

**Head**. In previous object detection algorithms, the head network often relied on fully connected layers or simple convolutional layers for object classification and localization, as depicted in (3). However, this conventional approach had limited capacity to capture complex patterns and fully leverage the rich image features. According to (4), we introduce attention-driven graph convolutional networks (AGCN) to enhance the performance of the head network. Eqs (5) and (6) represent the final outcomes for classification and regression. Fig 2(a) ⓒ illustrates the components of the head network, including Replicated Convolutional (RepConv), AGCN, and DETECT modules. The upper part of the diagram provides a specific schematic of the head network, while Fig 3 provides a detailed structural diagram to illustrate the individual roles and implementation details of each module.

RepConv introduce a parameter restructuring mechanism, which decomposes and recombines convolutional kernels to decouple the training and inference processes. This mechanism can reduce the computational and storage costs of the model to some extent, while also helping to improve the performance of object detection models.

A notable inclusion in the head is the attention-driven graph convolutional module (AGCN). This module integrates the content-aware attention module (CAAM) to enhance the focus on the target region while mitigating the influence of irrelevant information. Furthermore, it constructs a graph structure based on the extracted high-level features. By leveraging graph convolutional networks (GCN), the AGCN module effectively learns the spatial relationships between target objects in the graph. Through these relationships, the AGCN module is able to model the content-aware category representations generated by the CAAM, thereby forming static and dynamic graphs. The head framework, which incorporates the AGCN module, is visually illustrated in Fig 3.

**Separation**. The trained DynamicFocusNet algorithm is a valuable tool for object detection in target images. Through the integration of a modified cropping technique, known as the CP module, the algorithm accurately determines the coordinates and dimensions of the target object based on the output of the bounding box algorithm, as evidenced by (9), Figs 1(d) and 2(a) ⓓ. This integration enables the precise separation of detected objects from the original images, preserving important visual information and minimizing distortion. Consequently, the object-focused image data augmentation (OFIDA) facilitates high-quality one-to-many image data augmentation of samples, ensuring diversity and quantity of data that closely represents real-world scenarios. By reducing the risk of introducing unrealistic and misleading visual patterns that could confuse the model.

The object-focused image data augmentation algorithm is summarized in Algorithm 1.

### 4.2 Loss functions

In object-focused image data augmentation, classification and localization are two core sub-tasks. A variety of classification loss and box regression loss have been proposed in recent years. In this section, we will provide an overview of these loss functions, followed by our selection of the most suitable loss functions for DynamicFocusNet.

#### 4.2.1 Classification loss

To address class imbalance and optimize the classifier in object detectors, various classification loss functions have been proposed. These include Focal Loss, Quality Focal Loss, VariFocal Loss, and Poly Loss. Focal Loss effectively handles class imbalance, VariFocal Loss balances the importance of positive and negative samples, and Poly Loss adapts to different tasks and datasets. For DynamicFocusNet, we evaluated these advanced loss functions and ultimately chose VariFocal Loss as the optimal solution.

$$\begin{array}{c} L_{cls}=\{\begin{array}{cc} -q(q\;\text{log}(p)+(1-q)\;\text{log}(1-p)), & q>0\\ -\alpha p^{\gamma }\;\text{log}\left(1-p\right). & q=0 \end{array} \end{array}$$ (10)

where *p* denotes the predicted IoU-aware classification score (IACS) and *q* represents the objectness score.

#### 4.2.2 Box regression loss

The accuracy of object localization is ensured through box regression loss. Early works employed L1 loss for box regression, while more recent approaches introduced well-designed losses such as IoU-series los. Variants of IoU-series loss, including GIoU, DIoU, CIoU, and *α*-IoU, have shown effectiveness due to their alignment with evaluation metrics. In our study, we conducted experiments with GIoU, CIoU, and DIoU. CIoU, which considers factors like overlapping area, center point distance, and aspect ratio, was specifically applied in DynamicFocusNet. CIoU is defined as follows:

$$\begin{array}{c} L_{\text{box}}=1-\text{IoU}+\frac{\rho^{2}(\;b,\;b^{\text{gt}})}{c^{2}}+\alpha v. \end{array}$$ (11)

where *b* and *b*^*gt*^ represent the center points of the predicted box and the ground truth box, respectively, and *ρ* represents the Euclidean distance between the two center points. *c* denotes the diagonal distance of the smallest closed rectangle that can simultaneously contain the predicted box and the ground truth box. *α* is a weighting function, while *v* is used to measure the similarity of aspect ratios. When the aspect ratios of the ground truth box and the predicted box are closer, *v* becomes smaller.

#### 4.2.3 Object loss

Object loss was originally proposed in FCOS [38] with the aim of reducing the scores of low-quality bounding boxes, making them filterable in post-processing. Its application in YOLOX [39] has been proven to accelerate convergence. As an anchor-free framework, DynamicFocusNet also adopts object loss to further improve the accuracy of object detection.

$$\begin{array}{c} L_{obj}=\lambda_{obj}\sum_{i=0}^{S^{2}-1}\sum_{j=0}^{B-1}\left[1_{ij}^{obj}\right](-\text{log}\left({\hat{p}}_{ij}\right)+\lambda_{coord}\sum_{k\in x,y,w,h}{\left({\hat{t}}_{ij}^{k}-t_{ij}^{k}\right)}^{2}). \end{array}$$ (12)

where ${\hat{p}}_{ij}$ represents the predicted probability of whether the *j* − *th* bounding box in prediction *i* contains an object, $t_{ij}^{k}$ represents the true value of the *k*th coordinate for the *j*th bounding box in grid cell *i*, and ${\hat{t}}_{ij}^{k}$ represents its corresponding predicted value. The Iverson bracket function $\left[1_{ij}^{obj}\right]$ indicates whether the *j*th bounding box in prediction *i* contains an object and whether it is the prediction with the highest Intersection over Union (IoU). The hyperparameter λ_*coord*_ is used to balance the box regression loss and objectness classification loss, and λ_*obj*_ is a hyperparameter used to balance the number of positive.

In summary, the loss function of the OFIDA algorithm consists of three parts:

$$\begin{array}{c} L_{\text{OFIDA}\;}=L_{cls}+L_{\text{box}}+L_{obj}. \end{array}$$ (13)

where $L_{cls}$, $L_{\text{box}}$, and $L_{obj}$ represent classification loss, box regression loss, and object loss, respectively.

**Algorithm 1** Object-Focused Image Data Augmentation Algorithm

**Input**: One image or sequence of images **I** to be detected.

**Output**: The images **I**_*i*_ of each separated target category by (9).

 1. *Object classification scores c computation*.

$c=\frac{1}{2}(c_{\left(0\right)}+c_{\left(1\right)})$ ,

  where *c*_(0)_ and *c*_(1)_ are defined as in (3) and (4).

 2. *Regression vector* **H** *and candidate box* **b**_*i*_ *score S*_*i*_ *computation*.

  **H** = **Δb** + **b**_*i*_ = R(**b**_*i*_) ⊙ **b**_*i*_ + **b**_*i*_,

$S_{i}=\{\begin{array}{cc} S_{i}, & \text{IoU}(\text{m},{\text{b}}_{i})<N_{t}\\ S_{i}(1-\text{IoU}(\text{m},{\text{b}}_{i})). & \text{IoU}(\text{m},{\text{b}}_{i})⩾N_{t} \end{array}$

  where IoU is defined as in (8).

3. *Loss function of DynamicFocusNet*.

$L_{\text{OFIDA}}=L_{cls}+L_{\text{box}}+L_{obj}$ ,

 where $L_{cls}$, $L_{\text{box}}$, and $L_{obj}$ are defined as in (10), (11), and (12).

4. *Object separation* **I**_*i*_: *one-to-many* image data augmentation.

 **I**_*i*_ = CP(**I**, **t**_*i*_),

 where **t**_*i*_ is the target bounding boxes.

### 4.3 Lion optimizer

The Lion optimizer [40] is a recently developed optimization algorithm. In this paper, we integrate it into the target detection algorithm DynamicFocusNet. To provide a clearer understanding of the Lion algorithm, this section will elaborate on three aspects: operating principles, differences from previous algorithms, and specific reasons for its adoption.

#### 4.3.1 Operating principles

The Lion optimizer operates on the principle of simplicity and efficiency, relying solely on momentum without the need to simultaneously maintain first and second-order moments. This streamlined approach not only conserves memory resources but is particularly advantageous for training large-scale models with substantial batch sizes. Additionally, Lion generates updates in the form of element-wise binary operations, representing the optimization process as symbolic operations. This feature facilitates updates with larger norms, thereby enhancing the overall optimization process.

#### 4.3.2 Differences from previous algorithms

Memory efficiency, computational speed, and simplicity of hyperparameters are crucial metrics for evaluating the performance of optimization algorithms. In these aspects, the Lion optimizer demonstrates significant advantages compared to algorithms like AdamW and various adaptive optimizers that require storing first and second-order moments. It exhibits notable improvements in terms of memory requirements, computational speed, and the number of hyperparameters.

**Memory Efficiency**: Compared to algorithms like AdamW and various adaptive optimizers requiring storage of first and second-order moments, Lion significantly reduces memory requirements by relying solely on momentum. This becomes crucial when training large models with substantial batch sizes, such as ViT-B/16.

**Computational Speed**: Lion exhibits faster execution times (steps per second) compared to AdamW and Adafactor, with speed improvements ranging from 2% to 15%. The simplicity of Lion contributes to enhanced efficiency across various tasks, codebases, and hardware configurations.

**Simplicity of Hyperparameters**: In contrast to AdamW and Adafactor, Lion introduces fewer hyperparameters, streamlining the tuning process. The default values for Lion’s hyperparameters are discovered through a systematic programming search process, enhancing user-friendliness.

#### 4.3.3 Reasons for lion optimizer adoption

By incorporating the Lion optimizer into DynamicFocusNet, significant improvements in the performance of DynamicFocusNet for object detection tasks can be achieved through the following optimization strategies:

**Adaptive Learning Rate Adjustment**: Harnessing the characteristics of the Lion optimizer, dynamically adjust the learning rate size and step length based on the gradient situation of each parameter for adaptive learning rate tuning. The advantage lies in enhancing the efficiency of DynamicFocusNet during training, facilitating faster convergence, thereby bolstering the model’s accuracy.

**Momentum Acceleration**: Utilizing the momentum mechanism of the Lion optimizer to reduce oscillations and fluctuations in gradient updates, contributing to the enhanced stability of the DynamicFocusNet model. Appropriately adjusting the momentum parameter value of the Lion optimizer can accelerate the convergence speed of the DynamicFocusNet model, further optimizing the performance of object detection.

**Parameter Distribution Balancing**: Leveraging the Lion optimizer’s features to dynamically adjust gradients, mitigating issues related to excessively sparse or dense parameter settings. In DynamicFocusNet, judiciously configuring the parameters of the Lion optimizer adjusts the distribution of parameters, improving the model’s generalization ability and robustness.

## 5 Experiments

In this section, we present comprehensive evaluations of the object-focused image data augmentation (OFIDA) algorithm.

### 5.1 Experimental setup

**Dataset**. We use a diverse set of datasets for our experimental evaluation, including CIFAR10, CIFAR100, ImageNet, PASCAL VOC, CITYSCAPES, and MS-COCO 2017. These datasets were selected to cover a wide range of image recognition, semantic segmentation, and object detection tasks, providing a comprehensive assessment of our proposed approach.

**Implementation details**. The OFIDA algorithm was trained from scratch, and other methods that rely on pre-trained models obtained from online resources provided by the authors. We relied solely on the corresponding training data without any external pre-training or fine-tuning. This approach allowed us to assess the genuine performance of our model on the datasets. The experiments were conducted on a system comprising an Intel(R) CoreTM i9-10900X CPU @ 3.70GHz × 20, NVIDIA Quadro RTX 8000 GPU, 96GB memory, and Ubuntu 20.04 LTS 64-bit operating system. Detailed information about the specific parameters employed during the training process can be found in Table 1.

**Table 1:** Parameters setting.

Hyperparameter	Value
Input imagesize	640 x 640
Number of classes	80
Learning rate	(1, 1e-5, 1e-1)
Optimizer	Lion [40]
Batch size	32
Number of epochs	300
Feature pyramid layers	5
Detection layers	3
IoU threshold	(0, 0.1, 0.7)

**Evaluation index**. For the CIFAR10, CIFAR100, and ImageNet datasets, the Accuracy is employed to evaluate the algorithm’s performance in image classification. On the PASCAL VOC and CITYSCAPES datasets, the proposed OFIDA algorithm is evaluated for semantic segmentation using the mean Intersection over Union (mIoU). For the MS-COCO 2017 dataset, the performance of the proposed DynamicFocusNet algorithm is evaluated using multiple metrics. The mean average precision (mAP) provides an overall assessment of the algorithm’s precision across different Intersection over Union (IoU) thresholds. The average precision (AP) at an IoU of 0.5 (AP_50_) and AP at an IoU of 0.75 (AP_75_) specifically measure the algorithm’s precision at those IoU thresholds. Additionally, the frames per second (FPS) metric is used to evaluate the algorithm’s computational efficiency.

The Accuracy can be defined as:

$$\begin{array}{c} \text{Accuracy}=\frac{\text{Number}\;\text{of}\;\text{correctly}\;\text{classified}\;\text{samples}}{\text{Total}\;\text{number}\;\text{of}\;\text{samples}}. \end{array}$$ (14)

The mIoU can be defined as:

$$\begin{array}{c} \text{mIoU}=\frac{1}{N}\sum_{i=1}^{N}\frac{{\text{A}}_{i}\cap {\text{B}}_{i}}{{\text{A}}_{i}+{\text{B}}_{i}-\left({\text{A}}_{i}\cap {\text{B}}_{i}\right)}. \end{array}$$ (15)

where *N* represents the total number of samples or classes, while **A**_*i*_ and **B**_*i*_ refer to the predicted region and ground truth region, respectively, for the *i-th* sample or class.

The mAP is the average value of AP. AP (Average Precision) measures the average precision of the model across different levels of recall. The definitions of Precision and Recall are as follows:

$$\text{Precision}=\frac{TP}{TP+FP},$$ (16)

$$\begin{array}{c} \text{Recall}=\frac{TP}{TP+FN}, \end{array}$$ (17)

where *TP* represents true positives, *FP* represents false positives, and *FN* represents false negatives. The AP and mAP can be respectively defined as:

$$\begin{array}{c} \text{AP}=\sum_{i=1}^{n-1}(\frac{r_{i+1}-r_{i}}{r_{i+1}})P_{\text{inter}}\left(r_{i+1}\right), \end{array}$$ (18)

where *r*_1_, *r*_2_,…, *r*_*n*_ are the recalls corresponding to the first interpolated precision value in each interval. *P*_inter_ is the interpolated precision at the corresponding recall level *r*_*i*+1_ The summation is taken over the range from 1 to *n* − 1, where *n* is the number of recall levels.

$$\begin{array}{c} \text{mAP}=\frac{\sum_{i=1}^{k}{\text{AP}}_{i}}{k}. \end{array}$$ (19)

where *k* is the total number of classes.

### 5.2 Comparing OFIDA with state-of-the-art methods

In this section, based on the taxonomy presented in 2.1, we present detailed results for image classification and semantic segmentation. To validate the effectiveness of OFIDA across various computer vision tasks, we maintain consistency with the testing methodology outlined in previous image data augmentation approaches [14]. In the field of deep generative models, we opted to compare with the advanced StarGAN v2 model [13]. StarGAN v2 [13], an upgraded version of StarGAN [12], is dedicated to further enhancing the quality and diversity of multi-domain image translation. The model introduces a probabilistic generator and discriminator, along with an unbalanced feature alignment mechanism, significantly improving the quality and diversity of generated images. The incorporation of complementary sample generation further strengthens the model’s performance. StarGAN v2 has made notable strides in improving the quality and diversity of generated images, rendering it more suitable for practical applications and a broader range of image translation tasks.

#### 5.2.1 Visualization

**Fig 4:**
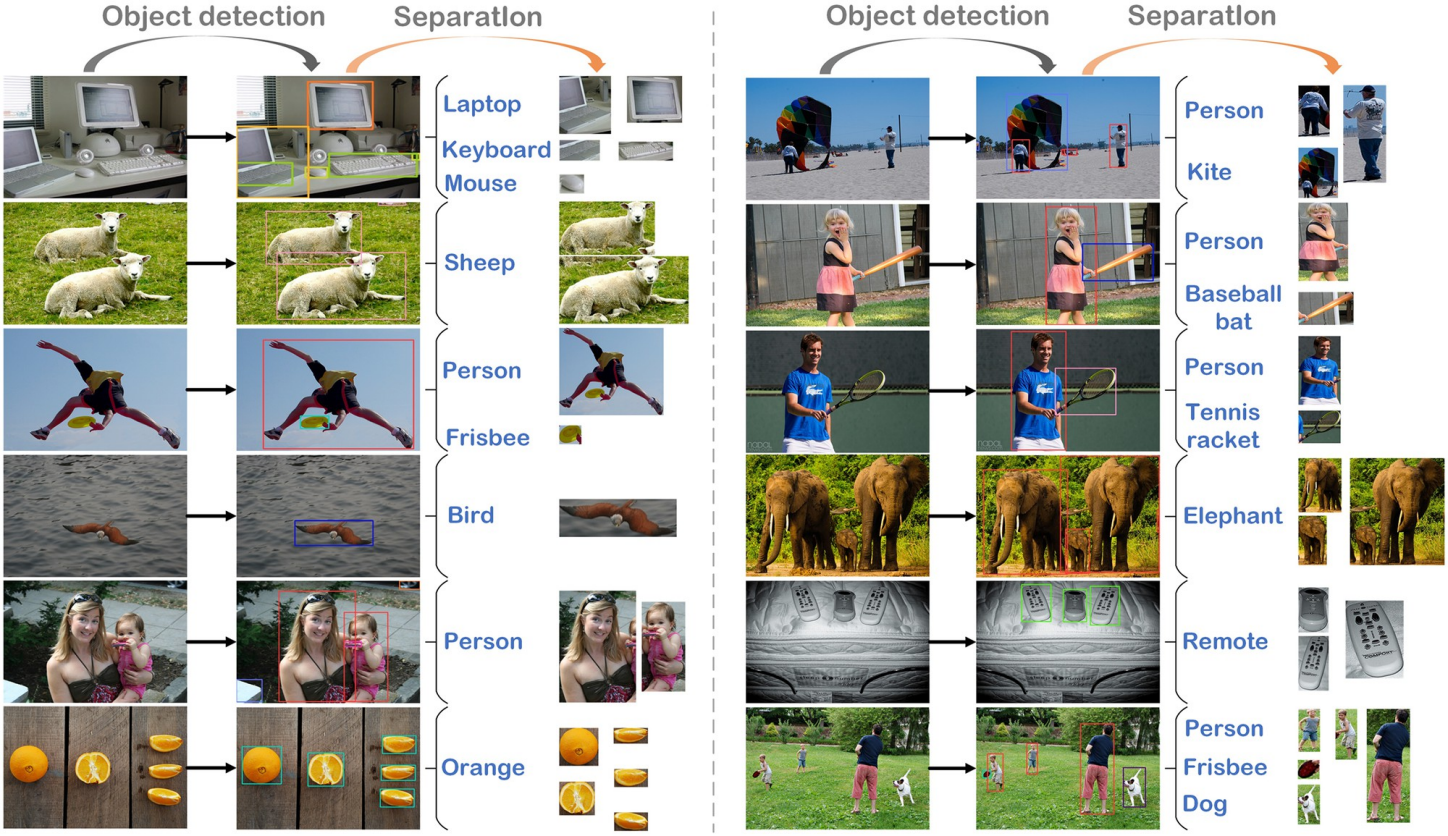
Visual examples of object-focused image data augmentation algorithm: Localization, classification, and separation of target regions from original images.

Visual examples of our proposed (OFIDA) algorithm are shown in Fig 4. This algorithm incorporates a localization, classification, and separation technique to effectively generate new training images, facilitating one-to-many data augmentation.

#### 5.2.2 Image classification

In this experiment, we compile and compare the results from the OFIDA and several state-of-the-art (SOTA) data augmentation methods, which are the same as those mentioned in Section 2.1. We compare the classification accuracy of various image classification techniques, including Wide-ResNet [41], DenseNet [42], and Shake ResNet [43], with and without data augmentation. The evaluation is performed on popular image classification datasets, namely CIFAR-10, CIFAR-100, and ImageNet.

Table 2 presents a summary of the image classification results obtained with and without data augmentation. It is evident that data augmentation leads to an average improvement in accuracy. Notably, the OFIDA algorithm achieves the highest accuracy among the data augmentation methods.

**Table 2:** Performance comparison of the OFIDA and several SOTA data augmentation methods for image classification.

Augmentation	CIFAR-10		CIFAR-100		ImageNet	
	Accuracy(%)	Model	Accuracy(%)	Model	Accuracy(%)	Model
**Baseline**	86.32	**Wide-ResNet** [41]	62.36	**DenseNet** [42]	74.12	**Shake ResNet** [43]
**image manipulation**	89.56	**Wide-ResNet** [41]	64.21	**DenseNet** [42]	77.02	**Shake ResNet** [43]
**image erasing**	93.24	**Wide-ResNet** [41]	67.34	**DenseNet** [42]	78.35	**Shake ResNet** [43]
**image mix**	92.36	**Wide-ResNet** [41]	66.51	**DenseNet** [42]	77.95	**Shake ResNet** [43]
**auto augment**	95.56	**Wide-ResNet** [41]	72.35	**DenseNet** [42]	79.56	**Shake ResNet** [43]
**feature augmentation**	95.89	**Wide-ResNet** [41]	73.21	**DenseNet** [42]	80.51	**Shake ResNet** [43]
**deep generative models** [13]	94.99	**Wide-ResNet** [41]	72.59	**DenseNet** [42]	80.23	**Shake ResNet** [43]
**OFIDA**	**96.54**	**Wide-ResNet** [41]	**80.55**	**DenseNet** [42]	**84.03**	**Shake ResNet** [43]

#### 5.2.3 Semantic segmentation

This subsection presents the results of semantic segmentation experiments conducted on the PASCAL VOC and CITYSCAPES datasets. To evaluate the effectiveness of the OFIDA algorithm and state-of-the-art (SOTA) data augmentation techniques in semantic segmentation tasks, we collected the validation set results on these datasets. The evaluation metric used is the mean Intersection over Union (mIoU), which represents the accuracy of the segmentation.

Tables 3 and 4 present the achieved mIoU scores on the PASCAL VOC dataset and the CITYSCAPES dataset. These tables include the results obtained by several semantic segmentation models, namely deeplabv3+ [44], PSPNet [45], GCNet [46], and ISANet [47]. The analysis reveals that the incorporation of data augmentation techniques enhances the performance of semantic segmentation models. Notably, the OFIDA algorithms demonstrate substantial improvements in performance compared to other techniques.

**Table 3:** Performance evaluation of semantic segmentation on the PASCAL VOC 2012 validation set using mIoU.

Augmentation	deeplabv3+ [44]	PSPNet [45]	GCNet [46]	ISANet [47]
**Baseline**	72.31	70.46	69.34	69.27
**image manipulation**	75.32	73.34	72.17	72.45
**image erasing**	74.89	73.12	71.86	71.37
**image mix**	76.24	74.34	73.57	73.30
**auto augment**	76.84	75.93	74.69	74.75
**feature augmentation**	75.93	74.94	72.37	72.71
**deep generative models** [13]	77.21	75.35	74.57	74.21
**OFIDA**	**79.86**	**78.02**	**76.96**	**76.34**

**Table 4:** Performance evaluation of semantic segmentation on the CITYSCAPES validation set using mIoU.

Augmentation	deeplabv3+ [44]	PSPNet [45]	GCNet [46]	ISANet [47]
**Baseline**	66.29	65.96	67.19	68.10
**image manipulation**	69.34	68.96	70.11	70.79
**image erasing**	68.78	68.43	69.68	70.28
**image mix**	70.19	69.85	71.08	71.94
**auto augment**	70.90	70.47	71.80	72.46
**feature augmentation**	69.46	69.16	70.39	71.11
**deep generative models** [13]	71.14	70.57	71.80	72.52
**OFIDA**	**73.86**	**73.18**	**74.51**	**75.05**

### 5.3 DynamicFocusNet performance evaluation

Our study primarily focused on exploring innovative data augmentation techniques, placing a higher emphasis on the accuracy performance of our model and its ability to accurately extract cropped target images from the original image after localization. Therefore, we did not prioritize the reduction of FLOPs (floating-point operations) or the number of parameters in our approach. Additionally, our secondary objective was to develop a model that could harness the capabilities of graph neural networks and overcome limitations of conventional object detection models, while achieving state-of-the-art accuracy.

Table 5 comprehensively presents the performance of DynamicFocusNet in terms of accuracy, speed, and robustness. Comparative results indicate that our approach strikes an ideal balance between speed and accuracy. DynamicFocusNet exhibits significant improvements compared to previous models such as YOLOR, YOLOv5, and YOLOX, achieving increases of 4.7%, 10.1%, and 8.6%, respectively, in average precision (AP). In comparison with PPYOLOE, which shares a similar inference speed, DynamicFocusNet demonstrates a noteworthy AP improvement of 6.5%. Despite the high inference speed of YOLOv6 and YOLOv7, DynamicFocusNet successfully boosts AP by 5.5% and 4.3%, respectively, while maintaining optimal efficiency. Notably, at a frame rate of 80 FPS, DynamicFocusNet achieves an AP of 55.5%, outperforming YOLOv8, which attains 53.9% AP at a frame rate of 68 FPS. Beyond the comprehensive AP evaluation, DynamicFocusNet excels in IoU threshold assessment (AP_50_ and AP_75_) and target size evaluation (AP_*S*_, AP_*M*_, and AP_*L*_), showcasing its broad applicability.

**Table 5:** DynamicFocusNet performance evaluation on MS-COCO 2017 val set.

Method	InputSize	MParams	GFLOPs	FPS	mAP	AP_50_	AP_75_	AP_*S*_	AP_*M*_	AP_*L*_
**YOLOR** [48]	640	53	120	82	50.8%	69.6%	55.7%	31.7%	55.3%	64.7%
**YOLOv5** [49]	640	21	49	94	45.4%	64.1%	48.9%	27.8%	50.4%	58.1%
**YOLOX** [39]	640	25	74	61	46.9%	65.6%	54.5%	29.8%	54.5%	64.4%
**PPYOLOE** [50]	640	23	50	92	49.0%	65.9%	53.0%	28.6%	52.9%	63.8%
**YOLOv6** [18]	640	35	86	111	50.0%	66.9%	54.3%	30.7%	55.2%	66.8%
**YOLOv7** [51]	640	37	105	123	51.2%	69.7%	55.5%	31.8%	55.5%	65.0%
**YOLOv8** [17]	640	68	258	68	53.9%	69.8%	58.5%	35.4%	59.1%	70.9%
**DynamicFocusNet**	640	116	323	80	**55.5%**	**70.2%**	**59.1%**	**36.8%**	**59.3%**	**71.5%**

## 6 Conclusion

The model and the algorithm for the problem of object-focused image data augmentation (OFIDA) have been investigated in this paper. Our contributions to this challenging problem are as follows:

**Model**: We form a novel model of the OFIDA problem to accurately identify and separate target regions in images while generating diverse and precise image samples to enable one-to-many data augmentation.

**Algorithm**: Based on the OFIDA model, an integrated algorithm which combines an optimized attention mechanism, a dynamic graph convolutional network (D-GCN), a novel object detection algorithm called DynamicFocusNet, and a modified cropping technique is presented as a solution to the OFIDA problem.

Numerical experiments were conducted to evaluate the performance of the proposed OFIDA algorithm. The results demonstrate that the OFIDA algorithm, through its ability to accurately classify, identify, and separate target images, and enable one-to-many data augmentation, significantly improves the performance of various computer vision tasks, such as image classification and semantic segmentation. Furthermore, the experimental findings highlight the superiority of the proposed DynamicFocusNet algorithm over other state-of-the-art object detection algorithms. In the future, it would be interesting to investigate how to further improve the accuracy and robustness of object detection. Additionally, considering the success of OFIDA in tasks like image classification and semantic segmentation, it becomes tempting to extend its application to other areas of computer vision, such as object tracking or scene understanding. Evaluating the performance of OFIDA in domains like medical imaging or remote sensing holds promise for new advancements and discoveries.
